# Cognitive enhancing effects of pazopanib in D‑galactose/ovariectomized Alzheimer’s rat model: insights into the role of RIPK1/RIPK3/MLKL necroptosis signaling pathway

**DOI:** 10.1007/s10787-023-01269-y

**Published:** 2023-07-17

**Authors:** Rasha Abdelhady, Nancy S. Younis, Omaima Ali, Samah Shehata, Rabab H. Sayed, Rania I. Nadeem

**Affiliations:** 1 Department of Pharmacology and Toxicology, Faculty of Pharmacy, Fayoum University, Fayoum, Egypt; 2 Department of Pharmaceutical Science, Faculty of Clinical Pharmacy, King Faisal University, Al Hofuf 31982, Al-Ahsa, Saudi Arabia; 3 Department of Biochemistry, Faculty of Pharmacy, Sinai University-Kantara Branch, Ismailia, 41636 Egypt; 4 General Division for Biological Control and Research, Egyptian Drug Authority, Cairo, 12618 Egypt; 5 Department of Biochemistry, Faculty of Pharmacy, Fayoum University, Fayoum, Egypt; 6 Department of Pharmacology and Toxicology, Faculty of Pharmacy, Cairo University, Kasr El-Aini Street, Cairo, Egypt; 7 Department of Pharmacology and Toxicology, Faculty of Pharmacy, Heliopolis University, Cairo, P.N. 11785 Egypt

**Keywords:** Alzheimer’s disease, D‑galactose, Ovariectomized, Pazopanib, Necroptosis, RIPK1, RIPK3, MLKL signaling

## Abstract

Necroptosis, a programmed form of necrotic cell death carried out by receptor-interacting serine/threonine protein kinase 1 (RIPK1) and RIPK3, has been found to be implicated in the pathogenesis of Alzheimer’s disease (AD). An FDA-approved anti-cancer drug, pazopanib, is reported to possess potent inhibitory effect against necroptosis via interfering with RIPK1. So far, there are no existing data on the influence of pazopanib on necroptotic pathway in AD. Thus, this study was designed to explore the impact of pazopanib on cognitive impairment provoked by ovariectomy (OVX) together with D-galactose (D-Gal) administration in rats and to scrutinize the putative signaling pathways underlying pazopanib-induced effects. Animals were allocated into four groups; the first and second groups were exposed to sham operation and administered normal saline and pazopanib (5 mg/kg/day, i.p.), respectively, for 6 weeks, while the third and fourth groups underwent OVX then were injected with D-Gal (150 mg/kg/day, i.p.); concomitantly with pazopanib in the fourth group for 6 weeks. Pazopanib ameliorated cognitive deficits as manifested by improved performance in the Morris water maze besides reversing the histological abnormalities. Pazopanib produced a significant decline in p-Tau and amyloid beta (Aβ) plaques. The neuroprotective effect of pazopanib was revealed by hampering neuroinflammation, mitigating neuronal death and suppressing RIPK1/RIPK3/MLKL necroptosis signaling pathway. Accordingly, hindering neuroinflammation and the necroptotic RIPK1/RIPK3/MLKL pathway could contribute to the neuroprotective effect of pazopanib in D-Gal/OVX rat model. Therefore, this study reveals pazopanib as a valuable therapeutic agent in AD that warrants future inspection to provide further data regarding its neuroprotective effect.

## Introduction

Alzheimer’s disease (AD) is the most common neurodegenerative disorder that impacts approximately 50 million individuals globally and it’s the sixth-leading cause of mortality in the United States (Alzheimer’s Association Report 2021). Pathologically, AD is distinguished by two hallmark lesions: intracytoplasmic neurofibrillary tangles (NFTs) and neuritic plaques. NFTs are intracytoplasmic deposits of the hyperphosphorylated microtubule-associated tau protein that is mislocalized in the brains of AD patients. While neuritic plaques are extracellular aggregates formed around a core of amyloid beta peptide (Aβ) derived from amyloid precursor protein (APP) (Buée et al. 2000; Serrano-Pozo et al. 2011; Khan and Bloom 2016). Other features of AD include neuroinflammation, cortical atrophy and significant neuronal loss. Clinically, AD is characterized by decline in recent memory function, disturbance in verbal fluency, visuospatial dysfunction and profound cognitive decline (Schultz et al. 2004; Weintraub et al. 2012; Deture and Dickson 2019). Molecular mechanisms underlying the neuronal loss in AD are still to be fully clarified.

Necroptosis is a newly identified cell death modality that is mainly characterized by mimicking feature of both necrosis and apoptosis and is usually triggered under apoptosis-deficient conditions (Dhuriya and Sharma 2018). In addition, necroptosis represents a mechanism of regulated necrotic cell death carried out by death receptors, and it is distinct from apoptosis in having a pro-inflammatory nature that is due to the release of cellular debris inducing inflammation (Degterev et al. 2005). In addition, necroptosis is defined as a death receptor-mediated extrinsic form of cell death that is morphologically significant from apoptosis (Degterev et al. 2008). Recent studies have started to investigate the potential role of necroptosis in the pathophysiology of several neurological disorders besides its relevance to other human diseases such as stroke, myocardial infarction and inflammatory disorders (Ofengeim and Yuan 2013; Weinlich et al. 2017). As far as this publication is concerned, no necroptotic inhibitors are currently being used in the therapeutic settings.

Recent researches elucidating the molecular mechanisms underlying necroptosis, documented that it is triggered by the activation of certain death receptors such as tumor necrosis factor receptor (TNFR) (Dhuriya and Sharma 2018). Stimulation of TNFR1 causes recruitment of TNFR1-associated death domain (DD) protein (TRADD) and receptor-interacting serine/threonine protein kinase 1 (RIPK1) (Degterev et al. 2008). Herein, dimerization and activation via ubiquitylation of RIPK1 take place where it represents the major signaling event in TNFR1 pathway. RIPK1 activation leads to the formation of a RIPK1–RIPK3–mixed lineage kinase-like protein (MLKL) complex. Notably, the formed MLKL complex is then phosphorylated by RIPK3 that triggers cell lysis via cell membrane disruption (Cho et al. 2009; Wang et al. 2014).

In addition to RIPK1 role as a key signaling protein in necroptosis cell death, it has a major function in inducing neuronal inflammation through RIPK1-mediated up-regulated expression of pro-inflammatory mediators like interleukin-1β (IL-1β) and TNF-α (Ito et al. 2016). Therefore, RIPK1-induced inflammatory response in microglia could potentially play a key role in the pathogenesis of AD that is characterized by chronic inflammation, elevated pro-inflammatory cytokines as well as microglial activation.

Consequently, previous evidences highlighted the contribution of necroptosis in the development and pathogenesis of AD (Mandrekar and Landreth 2010). Examination of postmortem samples of AD patients revealed significantly higher levels of RIPK1 in comparison to control ones and it was inversely proportional to brain weight as well as cognitive score (Caccamo et al. 2017; Ofengeim et al. 2017). Furthermore, a mouse model of AD has confirmed the association between RIPK1 and the development of AD. Firstly, elevated levels of RIPK1 were reported surrounding the characteristic Aβ plaques. Conversely, RIPK1 inhibition markedly reduced IL-1β and TNF-α levels, ameliorated the memory deficits as well as reduced Aβ burden (Ofengeim et al. 2017; Yang et al. 2017).

Pazopanib is an FDA-approved anti-cancer drug that is reported to be a potent necroptosis inhibitor via RIPK1 inhibition (Fauster et al. 2015). The ability of pazopanib to cross the blood brain barrier was reported by a previous study that also highlighted the beneficial effect of pazopanib treatment in modulating the inflammatory cytokines as well as decreasing p-tau protein level in a mouse model of tauopathies (Javidnia et al. 2017). Therefore, the present study aimed to investigate the possible neuroprotective role of pazopanib in AD rat model via modulating necroptosis.

## Materials and methods

### Animals

The current study involved 36 female adult, Wistar albino rats (140—180 g) purchased from the National Cancer Institute (Cairo, Egypt). Rats were housed (5/cage) through 12 h light/dark cycles at constant environmental conditions of 23 °C ± 2 and 50% ± 10 relative humidity. Rats were kept in the animal facility for 1 week before experiments for acclimatization. The protocol of this study was conducted in accordance with the ethical procedures and policies approved by the Ethics Committee for Animal Experimentation at Faculty of Pharmacy, Cairo University; PT (2991) and complied with the terms of the US National Institutes of Health guide for the Care and Use of Laboratory Animals (NIH Publication No. 85–23, revised 1996).

### Chemicals and drugs

Pazopanib was acquired from Novartis Healthcare Pvt. Ltd, INDIA, while D-galactose (D-Gal) was procured from Sigma-Aldrich Co, St. Louis, MO (USA). Fine chemicals and reagents were purchased from Sigma-Aldrich Chemical Co. with the highest analytical grade available, unless otherwise stated.

### Experimental design

Animals were randomly allocated into four groups (nine rats/group) as follow: first group (SO): sham operated animals represented the control group and received (2% Tween, 5% propylene glycol in 0.9% saline solution, i.p.) for 6 weeks; second group (Pazo): sham operated rats received pazopanib (5 mg/kg/day, i.p.) dissolved in (2% Tween, 5% propylene glycol in 0.9% saline solution) for 6 weeks (Javidnia et al. 2017); third group (D-Gal/OVX): rats were bilaterally ovariectomized (OVX), kept for 1 week, then injected intraperitoneally (i.p.) with D-Gal (150 mg/kg/day) dissolved in 0.9% saline solution for 6 weeks (Ibrahim et al. 2019); and fourth group (D-Gal/OVX + Pazo): D-Gal/OVX animals treated with pazopanib for 6 weeks starting 1 week after surgery (Table 1). The dose of pazopanib was selected based upon previous studies. One study found that pazopanib can restore memory loss and cognitive dysfunction to a similar extent as donepezil in a dosage of 15 mg/kg (Yang et al. 2015). On the other hand, another study showed that pazopanib in a dose of 10 mg/kg caused a significant biochemical and histopathological hepatotoxic effects in rats (Cetin et al. 2018). Moreover, a third study reported that pazopanib in a dose of 5 mg/kg crosses the blood–brain barrier with no detectable peripheral off-side effects, and decreases p-tau in an experimental model of tauopathy (Javidnia et al. 2017). Taken together, pazopanib in a dose of 5 mg/Kg was selected due to its brain penetrance ability, the reported non-detectable adverse effects associated with this dose and that 5 mg/Kg represents about $\frac{1}{16}$ of the equivalent clinical dosage used for cancer treatment (Nair and Jacob 2016) which minimizes any potential risk for toxicity or adverse effects.

**Table 1:** Experimental design

Animal group	Day 0	Day (1–7)	Day (8–49)	Morris water maze test	
				Day (45–48)	Day 49
SO	Sham operation	Healing and recovery	2% Tween, 5% propylene glycol in 0.9% saline, i.p	Memory acquisition training	Probe test
Pazo	Sham operation	Healing and recovery	Pazopanib (5 mg/kg/day, i.p.)	Memory acquisition training	Probe test
D-Gal/OVX	Two-sided ovariectomy	Healing and recovery	D-Galactose (150 mg/kg/day, i.p.)	Memory acquisition training	Probe test
D-Gal/OVX + Pazo	Two-sided ovariectomy	Healing and recovery	D-Galactose (150 mg/kg/day) + Pazopanib (5 mg/kg/day, i.p	Memory acquisition training	Probe test

*SO* sham operated, *Pazo* pazopanib, *D-Gal* D-galactose, *OVX* ovariectomized

### Surgery

Rats were anesthetized by i.p. injection of a mixture of ketamine hydrochloride (50 mg/kg) and xylazine (10 mg/kg), then a small incision between the hip and the last rib was made. Rat ovaries along with fat pads were externalized and a hemostatic lock was clenched around the blood supply to the ovaries creating a suture tangle beneath it. Afterward, the ovaries and fraction of the oviduct were detached, and the muscle and skin layers were stitched. Povidone iodine and bivatracin spray were applied topically for treating the wound. SO rats underwent all the aforementioned procedures except for the OVX. OVX rats received 0.1 mL diclofenac sodium and ceftriaxone (100 mg/ml) i.p. to accelerate the healing process (Khajuria et al. 2012). The experimental animals were fed on phytosteroid-free diet.

### Behavioral testing

Morris Water Maze (MWM) test tracked by ANYMAZE Software (Morris 1981) was adopted to assess learning ability and spatial memory. The maze is made of a stainless-steel circular tank (1.5 m diameter and 0.6 m height) which is divided into four quadrants and filled with water (25 ± 2^◦^ C) that is made opaque by a non-toxic colored dye. Within the target quadrant, a platform (0.1 m diameter and 0.28 m height) was submerged at 0.01 m underneath the water surface. Memory acquisition trials (120 s each) were accomplished for 4 consecutive days at the rate of two times/day, at least 5 min were left as a rest between the two trials. During each acquisition trial, animals were freely permitted to allocate the hidden platform inside the target quadrant. Once the rat could allocate the platform, it was allowed to rest there for an extra 20 s, and if a rat could not allocate the platform during 120 s, it was quietly directed to the platform and kept there for 20 s. On day 5, the animals were assessed through a probe-trial session through which the hidden platform was dislodged from the tank, and every single animal has been given 60 s to explore the tank. Time consumed by each rat inside the targeted quadrant represents a measurement for the recall memory.

### Brain processing

After performing the MWM test, animals were euthanized by cervical dislocation under thiopental anesthesia (5 mg/Kg). Each group was then subdivided into two subsets. Brains of the first subset (n = 3) were rapidly isolated and kept in 10% formalin to perform histopathological analysis. The hippocampi of the other subset (n = 6) were rapidly removed, weighed and stored at -80^◦^ C for biochemical investigations.

### Histopathological examination

For estimation of neuronal damage in hippocampal tissues, hematoxylin and eosin stain (H&E) and Nissl staining were performed. After fixing brain tissues for 72 h in neutral buffered formalin (10%), samples were processed with ethanol (sequential grades), cleared in xylene then infiltrated and fixed in Paraplast tissue embedding media. For demonstrating hippocampal regions, brain serial coronal Sects. (4-μm) were cut by rotatory microtome and mounted on glass slides. Tissue staining with H&E was used for light microscopic examination, whereas toluidine blue staining was used for determination of mean intact neurons count (Culling 1974).

For p-Tau immunohistochemistry, paraffin-embedded tissue sections were set according to the manufacturer’s instructions. Deparaffinized sections were processed by 0.3% H_2_O_2_ for 20 min then incubated overnight with anti-phospho Tau (1:100, Thermo Fisher scientific, Rockford, IL, USA, Cat No. 44-742G) at 4 °C. PBS was utilized to wash the tissue sections before being incubated for 20 min with secondary antibody HRP Envision kit (DAKO); then washed out again before being further incubated for 15 min with diaminobenzidine (DAB). Finally, washed by PBS, counter stained with hematoxylin, dehydrated, and cleared in xylene then cover slipped to be examined microscopically. For each sample, six non-overlapping fields were chosen at random and scanned from hippocampal CA3 region to assess the area percentage of immunohistochemical p-Tau expression levels in immunohistochemically stained sections as well as the mean count of intact neurons in toluidine blue stained tissue sections (Gad et al. 2022). Leica Application module for histological analysis linked to Full HD microscopic imaging system (Leica Microsystems GmbH, Germany) was employed for gathering all microscopic examination and data.

### Biochemical investigation

#### ELISA assessment

Hippocampi were homogenized in ice-cold PBS to yield 10% homogenates and the Rat ELISA specific commercial kits were utilized according to the manufacturer’s protocol to assess the tissue contents of the following biomarkers: TNF-α (Cat. No. MBS2507393, MyBioSource, San Diego, CA, USA), IL-1β (Cat. No. ELR-IL1b-CL, Ray Biotech, Norcross, USA), Aβ1-42 (Cat. No. MBS726579, MyBioSource, San Diego, CA, USA) and nuclear factor kappa B p65 (NF-ĸb p65) (Cat. No. E-EL-R0674, ElabScience, Biotechnology Co., Ltd, USA). All results of measured parameters were presented as pg/mg of protein.

#### Western blot analysis

Bio-Rad Mini-Protein II system was applied to discrete protein contents of dissected hippocampal tissues according to their molecular weight by equally loading them on sodium dodecyl sulfate–polyacrylamide gel electrophoresis (SDS-PAGE) lane. A Trans-Blot® Turbo™ Transfer System (Bio-Rad Laboratories GmbH, München, Germany) was employed to transfer proteins to a polyvinylidene difluoride membrane. In order to block nonspecific binding sites, membranes were soaked in tris-buffered saline + Tween 20 + 3% bovine serum albumin at room temperature for one hour. Next, the blots were developed through overnight incubation at 4 °C with antibodies (1:1000 dilution) against p-RIPK1(Ser 166) (Cat. No. 28252–1-AP), p-RIPK3 (Ser 232) (Cat. No. PA5-105701), β-actin (Cat. No. MA5-15739); obtained from Thermo Fisher Scientific Inc. (Rockford, IL, USA) and anti p-MLKL (Ser 358) (Cat. No. ab 187091; Abcam, UK). The blots were washed various times in Trisbuffered saline and Tween 20 before incubation with horseradish peroxidase-conjugated secondary antibody (Dianova, Hamburg, Germany). Lastly, protein bands were obtained via an enhanced chemiluminescence substrate reaction (Amersham Biosciences, Arlington Heights, IL, USA). The protein bands intensities were assessed against the control sample by means of densitometric analysis through using a scanning laser densitometer (Biomed Instrument, Inc., CA, USA). The results were presented in the form of arbitrary units.

#### Quantitative real-time polymerase chain reaction (RT-PCR)

RNeasy Mini Kit (QIAGEN; Cat. No. 74106) was used in accordance with the manufacturer's procedures to extract total RNA from rat hippocampal tissues. Then, Standard Chip of the Bio Rad Experion Automated Electrophoresis System (Bio-Rad Laboratories) was applied to examine the quality and integrity of extracted RNA. Spectrophotometry was then applied to settle the extracted RNA quantity. Subsequently, SuperScript III First-Strand Synthesis System (Cat. No. K1621; Fermentas, Waltham, MA, USA) was utilized in accordance with the manufacturer's steps to synthesize cDNA from 1 μg RNA. An applied Biosystem with software version 3.1 (StepOne™, USA) was used for RT-PCR amplification and analysis. The reaction had SYBR Green Master Mix (Applied Biosystems) and gene-specific primer pairs (shown in Table 2). Gene Runner Software (Hasting Software, Inc., Hasting, NY) was utilized to design gene-specific primer pairs from RNA sequences of the gene bank. Each set of primers showed 60° C calculated annealing temperature. RT-PCR was carried out in a 25-μl reaction volume containing 2X SYBR Green PCR Master Mix (Applied Biosystems), 900 nM of each individual primer and 2 μl of cDNA. Settings for amplification process were as follow: 50° C for 2 min, 95° C for 10 min, 40 cycles of denaturation for 15 s and annealing/extension at 60° C for 10 min. The v1·7 sequence detection software from PE Biosystems (Foster City, CA) was used to calculate all data recruited from real-time assays. The 2^−ΔΔCT^ formula was applied to calculate the relative expression of the target gene where whole values were standardized to the levels of β-actin and expressed as fold changes.

**Table 2:** Primer sequence of the examined genes

Gene	Primers
TNFR1	5′-GAC TGG TTC CTT CTC TTG GT 3′-GGT GTT CTG TTT CTC CTT AC
β-actin	F: 5′-TGCTGGTGCTGAGTATGTCG-3′ R: 5′P-TTGAGAGCAATGCCAGCC-3′

*TNFR1* tumor necrosis factor receptor1

### Statistical analyses

All statistical analyses were performed by Graph Pad Prism 9 software utilizing *P* value < 0.05 as the criterion for significance. Data were represented as mean ± SD. One-way analysis of variance (ANOVA) test followed by Tukey's multiple comparisons post-test was preformed to estimate the significant difference among the experimental groups.

## Results

Noteworthy, the administration of pazopanib to SO rats did not produce any significant difference in the whole set of evaluated parameters.

### Effect of pazopanib on D-Gal/OVX-induced behavioral changes in the MWM test

**Fig. 1:**
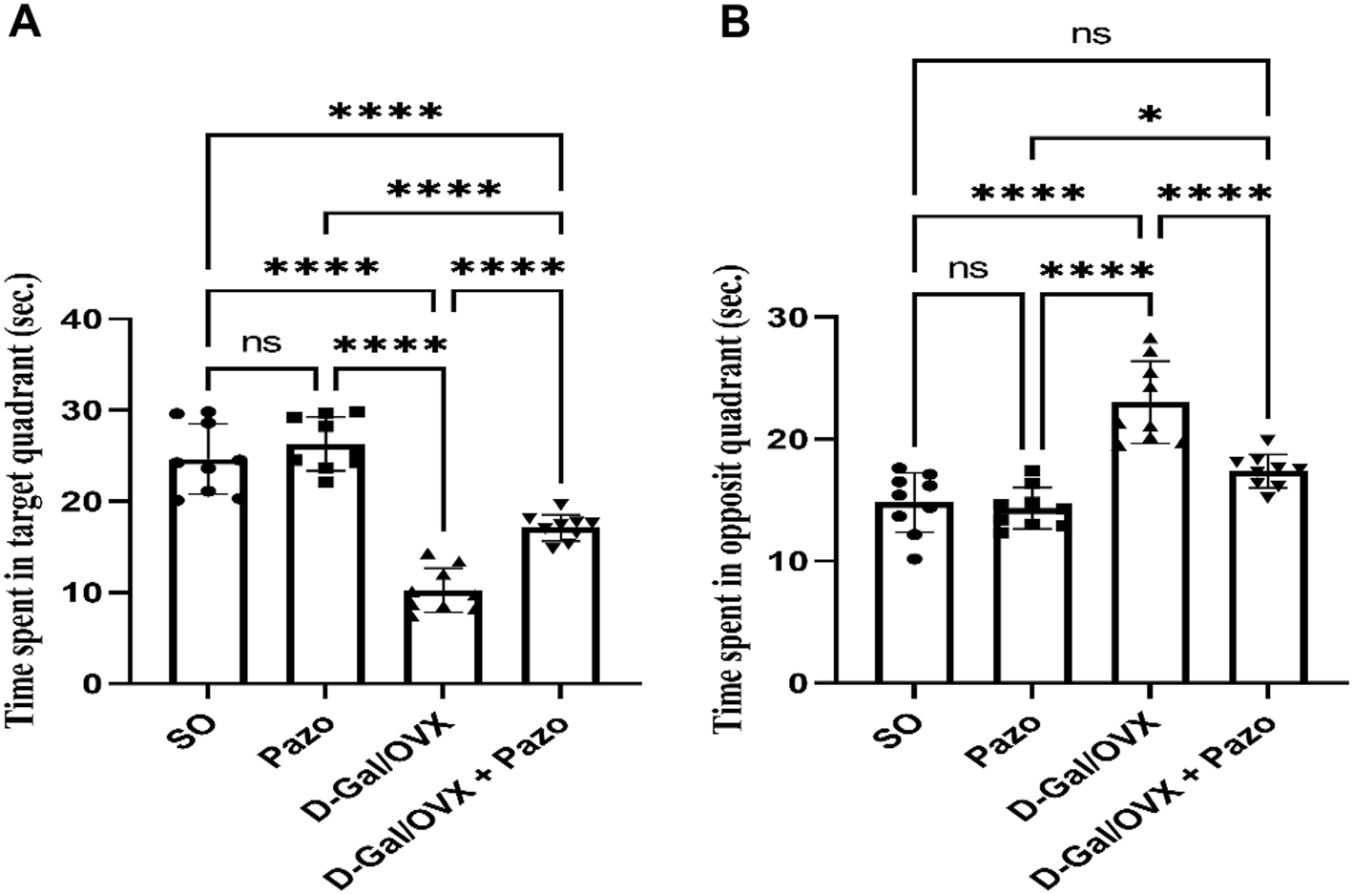
Effect of pazopanib on D-Gal/OVX-induced behavioral changes in the Morris water maze test: **A** time spent in target quadrant and **B** time spent in opposite quadrant. Values are presented as mean ± SD (*n* = 9). Statistical investigations were accomplished by One-way analysis of variance (ANOVA) followed by Tukey’s multiple comparisons test. *p < 0.05, **p < 0.01, ***p < 0.005, ****p < 0.001, *ns* no significance, *SO* sham operated, *Pazo* pazopanib, *D-Gal* D-galactose, *OVX* ovariectomized

The memory acquisition test revealed that the D-Gal/OVX group showed a significant decline in the time spent in the target quadrant and a significant elevation in the time spent in the opposite quadrant by approximately 41% and 1.6-fold, respectively, as compared with the SO group values. However, D-Gal/OVX + Pazo group showed higher records for the time spent in the target quadrant as well as lower ones for the time spent in the opposite quadrant by approximately 1.7- fold and 24.5%, respectively, as compared with the D-Gal/OVX group values (*F* (3, 32) = 61.99 and 25.82, respectively, *P* < 0.0001). (Fig. 1).

### Effect of pazopanib on D-Gal/OVX-induced histopathological changes

**Fig. 2:**
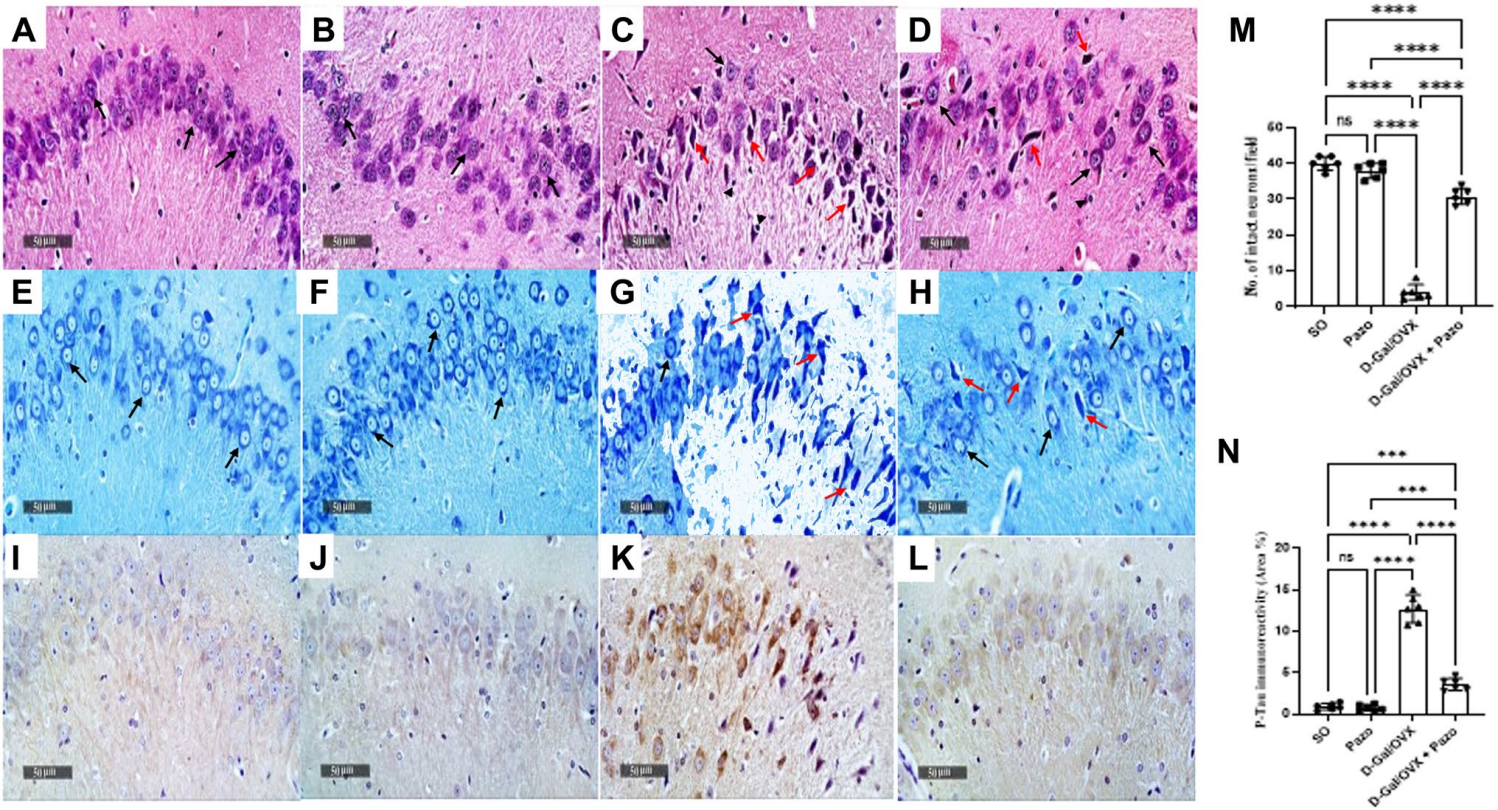
Effect of pazopanib on D-Gal/OVX-induced histopathological changes. **A**–**D** hippocampal sections stained with H&E. **A** and **B** The SO and Pazo groups, respectively, showing well organized histological features of CA3 layers with multiple apparent intact pyramidal neurons (black arrow) and intact intercellular brain matrix as well as minimal glial cells infiltrates. **C** D-Gal/OVX group showed scattered few apparent intact cells, severe diffuse neuronal degenerative patterns, many figures of hyperesenophilic and pyknotic neurons missing their subcellular (red arrow), brain matrix appeared to be moderately edematous with moderate higher records of reactive glial cells infiltrates (arrow head). **D** The D-Gal/OVX + Pazo group exhibited marked neuroprotection with significant higher numbers of intact neurons and more organized histological features along with mild reactive glial cells infiltrates. **E**–**H** hippocampal sections stained with toluidine blue for determination of the intact neurons count. **E** SO. **F** Pazo. **G** D-Gal/OVX. **H** D-Gal/OVX + Pazo. **I**–**L** Sections for the determination of P-Tau immunoexpression in hippocampal CA3 region. **I** SO. **J** Pazo. **K** D-Gal/OVX. **L** D-Gal/OVX + Pazo. **M** Intact neurons count in the CA3 region. **N** area percentage of P-Tau immunoreactivity in hippocampal CA3 region of the four groups. Values are presented as mean ± SD (*n* = 3). Statistical investigations were accomplished by One-way analysis of variance (ANOVA) followed by Tukey’s multiple comparisons test. *p < 0.05, **p < 0.01, ***p < 0.005, ****p < 0.001, *ns* no significance, *SO* sham operated, *Pazo* pazopanib, *D-Gal* D-galactose, *OVX* ovariectomized

Microscopic examination of the hippocampal CA3 region in the SO and pazopanib group samples demonstrated normal histological features (Fig. 2A and B). However, the D-Gal/OVX group samples exhibited severe neuronal necrosis, extensive pyknosis, cellular oedema and reactive glial cells infiltrates (Fig. 2C). Interestingly, sections from rats treated with pazopanib showed apparently intact neurons with minimal scattered records of degenerated neurons and mild reactive glial cells infiltrates (Fig. 2D).

Sections from D-Gal/OVX group also showed decreased number of intact neurons in the CA3 hippocampal subregion by 90% as compared with SO group (Fig. 2G and M) (*F* (3, 20) = 381.4, *P* < 0.0001). However, treatment with pazopanib reversed this deterioration showing a sevenfold increase in intact neuron count (Fig. 2H and M).

Hippocampal CA3 sections from SO rats showed homogenous P-Tau immunoexpression with few positive cells (Fig. 2I and J). On the other hand, the D-Gal/OVX samples revealed a significant increase in P-Tau immunoexpression by 13.8-fold as compared with the SO group (Fig. 2K and N). Pazopanib treatment mitigated this effect as evidenced by significantly lowered P-Tau immunoexpression by 71.7% as compared with D-Gal/OVX group (Fig. 2 L and N) (*F* (3, 20) = 213.9, *P* < *0.0001).*

### Effect of pazopanib on D-Gal/OVX-induced changes in hippocampal level of inflammatory markers and Aβ1-42

**Fig. 3:**
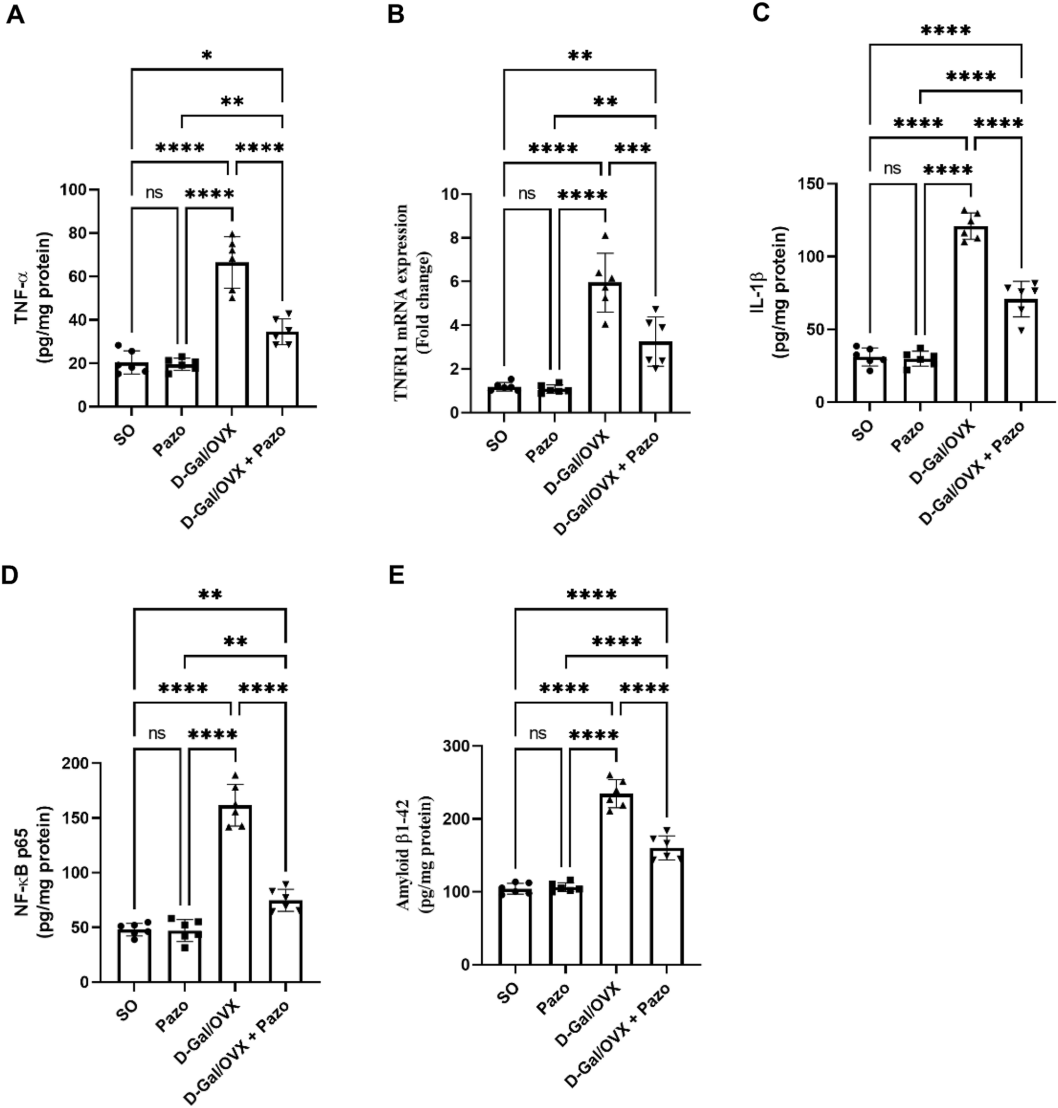
Effect of pazopanib on D-Gal/OVX-induced changes in hippocampal TNF-α (**A**), TNFR1 mRNA expression (**B**), IL-1β (**C**), NF-ĸB p65 (**D**), and Aβ1-42 (**E**). Values are presented as mean ± SD (*n* = 6). Statistical investigations were accomplished by One-way analysis of variance (ANOVA) followed by Tukey’s multiple comparisons test. *p < 0.05, **p < 0.01, ***p < 0.005, ****p < 0.001, *ns* no significance, *SO* sham operated, *Pazo* pazopanib, *D-Gal* D-galactose, *OVX* ovariectomized, *TNF-α* tumor necrosis factor-alpha, *TNFR1* tumor necrosis factor receptor1, *IL-1β* interlukin-1 beta, *NF-κB p65* nuclear factor kappa B p65

As shown in Fig. 3, OVX with D-Gal administration significantly augmented the level of TNF-α, TNFR-1 mRNA expression, IL-1β, NF-ĸB p65 and Aβ1-42 levels in the hippocampal tissues by approximately 3.2-, 5-, 3.9-, 3.4- and 2.2-folds, respectively, as compared with the SO group values. However, treatment with pazopanib showed an anti-inflammatory activity as evidenced by significant reduction of TNF-α level, TNFR-1 mRNA expression, IL-1β, NF-ĸB p65 and Aβ1-42 levels by approximately 48%, 45%, 41.5%, 53.8% and 31.8% as compared with the D-Gal/OVX group values, respectively (*F* (3,20) = 54.15, 39.87, 149.2, 117.3 and 123.0, respectively, *P* < *0.0001*).

### Effect of pazopanib on D-Gal/OVX-provoked changes of necroptotic markers

**Fig.4:**
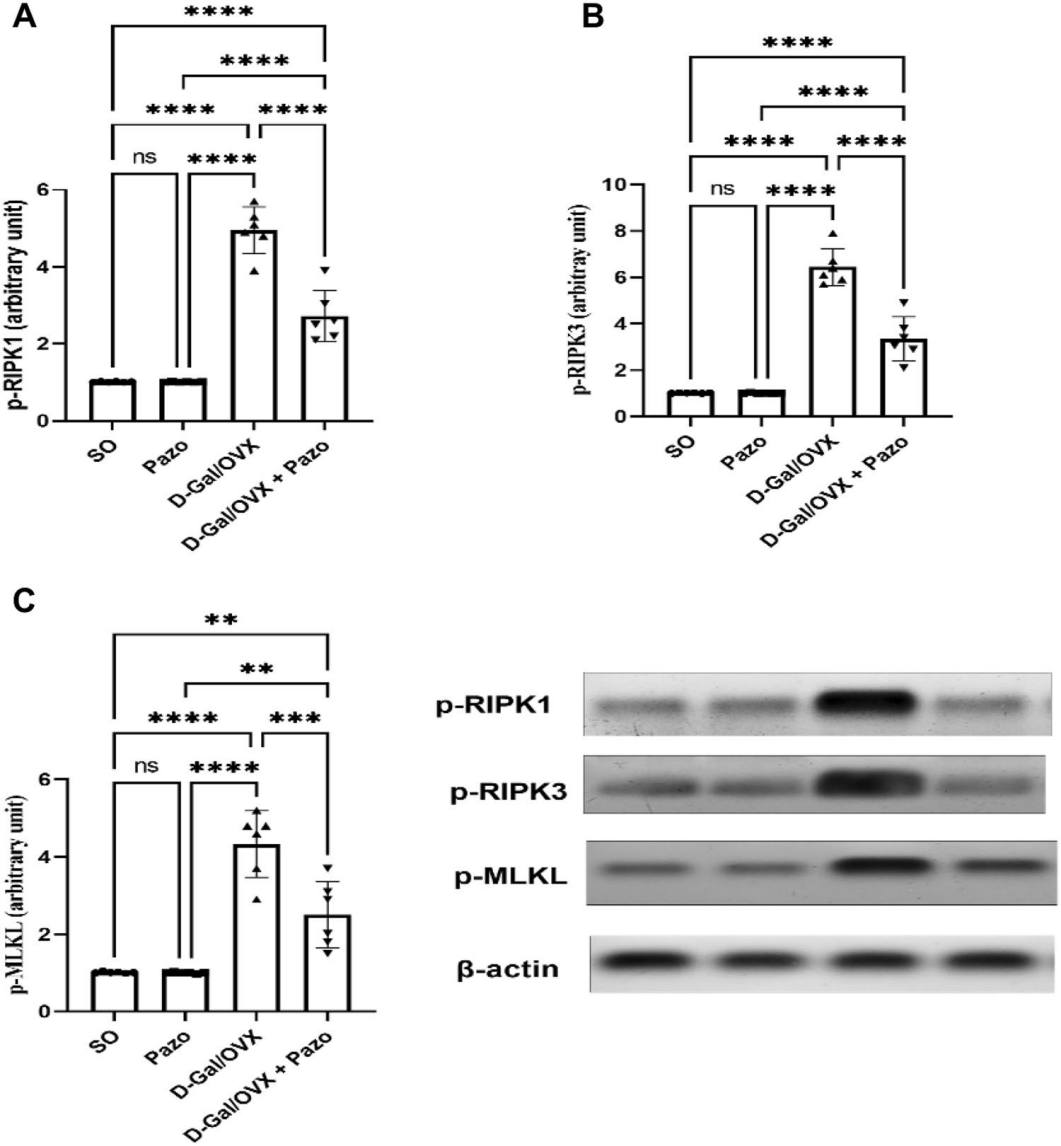
Effect of pazopanib on D-Gal/OVX-provoked changes in p-RIPK1 (**A**), P-RIPK3 (**B**), and p-MLKL (**C**) expression. Values are presented as mean ± SD (*n* = 6). Statistical investigations were accomplished by One-way analysis of variance (ANOVA) followed by Tukey’s multiple comparisons test. *p < 0.05, **p < 0.01, ***p < 0.005, ****p < 0.001, *ns* no significance, *SO* sham operated, *Pazo* pazopanib, *D-Gal* D-galactose, *OVX* ovariectomized, *p-RIPK1* phosphorylated receptor-interacting serine/threonine protein kinase 1, *p-RIPK3* phosphorylated receptor-interacting serine/threonine protein kinase 3, *p-MLKL* phosphorylated mixed lineage kinase-like protein

The data presented in Fig. 4 demonstrate that OVX with D-Gal injection provoked a significant elevation in p-RIPK1, p-RIPK3, and p-MLKL by approximately 4.9-, 6.3-, and 4.3-folds, respectively, as compared to SO group (*F* (3, 20) = 103.5, 104.57, and 40.44, respectively, *P* < 0.0001). In contrast, the pazopanib-treated rats showed a reduction in p-RIPK1, p-RIPK3, and p-MLKL by 45%, 48%, and 42%, as compared with D-Gal/OVX animals.

## Discussion

As far as we can tell, this is the first study to reveal the neuroprotective effect of pazopanib, an FDA-approved anti-cancer drug, against D-Gal/OVX-induced experimental AD model in rats. This conception is corroborated by (i) alleviation of spatial memory deficits together with modulation of the histopathological changes, (ii) decrement of AD hallmarks (p-Tau and Aβ plaques), (iii) suppression of the necroptotic p-RIPK1/p-RIPK3/p-MLKL signaling pathway, and (iv) mitigation of neuroinflammation as well as neuronal death.

Long-term D-Gal administration along with OVX represents an excellent experimental model for AD simulating the behavioral, neurochemical, and pathological manifestations detected in AD (Hua et al. 2007). Indeed, various studies have successfully utilized this model to induce AD-like features in experimental animals (Ibrahim et al. 2019, 2022; Abd El-Rahman and Fayed 2022). In the present investigation, D-Gal/OVX rats demonstrated marked cognitive deterioration and memory dysfunction manifested via their performance in the MWM test and the observed histological alterations. These outcomes are in good agreement with preceding results (Ibrahim et al. 2020; Ibrahim et al. 2022). Instead, pazopanib amended the cognitive functions which is substantiated by the histopathological improvement. Similarly, a former study reported that pazopanib reversed the spatial memory deficits in the MWM test and enhanced the brain function in a rat model of neurodegeneration induced by quinolinic acid (Yang et al. 2015).

Neuritic plaques of Aβ protein and NFTs with hyper p-Tau protein are the two hallmark proteins of AD. These two pathological features contribute to synaptic dysfunction, mitochondrial injury, microglial activation and neuroinflammation, ultimately leading to neuronal death (Zhang et al. 2021). In the current work, a substantial increase in Aβ1-42 level and p-Tau immunoexpression were identified in D-Gal/OVX animals which is in line with earlier findings (Ibrahim et al. 2019, 2020). On the other hand, pazopanib curbed such increment of both Aβ1-42 and p-Tau expression. Interestingly, our results are in partial agreement with a previous literature which evaluated the influence of pazopanib in two transgenic mouse models. It showed that pazopanib reduced p-Tau levels in TauP301L mice, transgenic mice overexpressing human tau, but did not affect Aβ concentration in 3x-AβPP mice, transgenic mice overexpressing Aβ protein precursor (Javidnia et al. 2017). This partial discrepancy may be attributed to the variation in the species as well as the experimental animal model used. Notably, Aβ neurotoxicity involves triggering the phosphorylation of tau protein (Wang et al. 2016; Zhang et al. 2021). Thus, the detected decline of p-Tau by pazopanib could be probably ascribed to its suppressive action on Aβ.

Various studies have shed the light on the association of necroptosis with neurodegenerative and neuroinflammatory disorders, like multiple sclerosis (Ofengeim et al. 2015), AD (Caccamo et al. 2017) and amyotrophic lateral sclerosis (Re et al. 2014). Caccamo et al. revealed that necroptosis is triggered in the brains of AD patients and is positively linked to neuronal death and neuroinflammation (Caccamo et al. 2017). In line, necrostatin‐1, an anti‐necroptotic molecule, was found to ameliorate cognitive impairment in AD models through targeting Aβ and tau proteins plus alleviating brain cell death (Yang et al. 2017). Necroptosis can be activated via the stimulation of certain death receptors including TNFR1 with its respective agonist TNFα (Yuan et al. 2019). This TNFα/TNFR1 interaction, under certain conditions, causes activation and phosphorylation of RIPK1. Accordingly, p-RIPK1 interacts with RIPK3 inducing its phosphorylation which in turn activates and phosphorylates MLKL causing its oligomerization. Thereafter, p-MLKL migrates to the plasma membrane, destabilizes it causing cell lysis and necroptotic cell death (Jayaraman et al. 2021). The present results showed that D-Gal/OVX rats exhibited a marked rise in TNFR1, p-RIPK1, p-RIPK3 and p-MLKL expression together with TNFα level suggesting the stimulation of TNFR1-mediated necroptotic pathway. In contrast, pazopanib abrogated the D-Gal/OVX-induced increment in the necroptotic markers expression causing a prompt decline in their level. In this regard, a previous study identified pazopanib as a necroptotic inhibitor impeding the phosphorylation of MLKL via targeting RIPK1 in human cells (Fauster et al. 2015).

Inflammation is a major culprit involved in the pathophysiology of AD. Neuroinflammation is provoked by the Aβ-induced instigation of microglia and astrocytes, where they secrete a profusion of pro-inflammatory cytokines (Glass et al. 2010). These cytokines alter the neurogenesis and neuronal plasticity contributing to neuronal loss (Rubio-Perez and Morillas-Ruiz 2012). Interestingly, RIPK1, the critical mediator of necroptotic cell death, has also been shown to promote neuroinflammation through the production of pro-inflammatory cytokines (Christofferson et al. 2012; Najjar et al. 2016; Ofengeim et al. 2017). Moreover, activated necrotic cell death can boost inflammation through the passive leakage of intracellular content as a consequence of disrupted cytoplasmic membrane (Ofengeim and Yuan 2013). Hence, RIPK1-mediated inflammatory response could potentially trigger the NF-κB signaling boosting the release of pro-inflammatory cytokines which consequently aggravates the pathogenesis of AD via a malicious loop of inflammation. Herein, pazopanib impeded the D-Gal/OVX-induced surge in the levels of TNF-α and IL-1β reflecting its anti-inflammatory effect. Additionally, pazopanib hindered the NF-κB p65 content detected in the hippocampus of D-Gal/OVX rats, with its subsequent pathologic actions, a result which confirms its anti-inflammatory property. In context, pazopanib was reported to restore inflammatory markers in 3x-AβPP mice (Javidnia et al. 2017). Also, another study stated that pazopanib can halt rat liver fibrosis through modulating the pro-inflammatory cytokines TNF-α and IL-6 (Elshal et al. 2015). Furthermore, a study conducted on patients with renal cell carcinoma revealed that pazopanib reduced the NF-κB p65 level in kidney cancer tissues (Spirina et al. 2017). NF-κB activation triggers the transcription of β-secretase (Tamagno et al. 2012) as well as several pro-inflammatory genes such as TNF-α (Lu et al. 2010). Besides, it diminishes microglial cells’ ability to phagocytose Aβ42 peptide monomers, leading to their aggregation into higher order amyloid structures (Zhao et al. 2014). Consequently, the inhibitory effect of pazopanib on NF-κB could participate in its neuroprotective impact against AD.

## Conclusion

In conclusion, the present findings demonstrate that treatment with pazopanib amended the cognitive functions, modulated the AD-associated histopathological alterations, diminished the AD hallmarks (p-Tau and Aβ plaques), and mitigated neuroinflammation in D-Gal/OVX rat model of AD. These favorable effects could be probably mediated via suppressing the necroptotic p-RIPK1/p-RIPK3/p-MLKL signaling pathway. Therefore, this study reveals pazopanib as a valuable therapeutic agent in AD that warrants future inspection to provide further data regarding its neuroprotective effect.

Limitations of the study include lack of data about potential side effects, full dose-responses, data on bioavailability and chemical purity of pazopanib.

## Data Availability

The datasets generated and/or analyzed during the current study are available from the corresponding author on reasonable request.
